# Automatic Prediction of Facial Trait Judgments: Appearance vs. Structural Models

**DOI:** 10.1371/journal.pone.0023323

**Published:** 2011-08-17

**Authors:** Mario Rojas Q., David Masip, Alexander Todorov, Jordi Vitria

**Affiliations:** 1 Computer Vision Center, Edifici O, Campus Bellaterra, Universidad Autonoma de Barcelona, Barcelona, Spain; 2 Departament of Estudis d'Informatica, Multimedia i Telecomunicacio, Universidad Oberta de Catalunya, Barcelona, Spain; 3 Department of Psychology, Princeton University, Princeton, New Jersey, United States of America; 4 Department de Matematica Aplicada i Analisi, Universitat de Barcelona, Barcelona, Spain; National Institute of Mental Health, United States of America

## Abstract

Evaluating other individuals with respect to personality characteristics plays a crucial role in human relations and it is the focus of attention for research in diverse fields such as psychology and interactive computer systems. In psychology, face perception has been recognized as a key component of this evaluation system. Multiple studies suggest that observers use face information to infer personality characteristics. Interactive computer systems are trying to take advantage of these findings and apply them to increase the natural aspect of interaction and to improve the performance of interactive computer systems. Here, we experimentally test whether the automatic prediction of facial trait judgments (e.g. dominance) can be made by using the full appearance information of the face and whether a reduced representation of its structure is sufficient. We evaluate two separate approaches: a holistic representation model using the facial appearance information and a structural model constructed from the relations among facial salient points. State of the art machine learning methods are applied to a) derive a facial trait judgment model from training data and b) predict a facial trait value for any face. Furthermore, we address the issue of whether there are specific structural relations among facial points that predict perception of facial traits. Experimental results over a set of labeled data (9 different trait evaluations) and classification rules (4 rules) suggest that a) prediction of perception of facial traits is learnable by both holistic and structural approaches; b) the most reliable prediction of facial trait judgments is obtained by certain type of holistic descriptions of the face appearance; and c) for some traits such as attractiveness and extroversion, there are relationships between specific structural features and social perceptions.

## Introduction

There is a long tradition of research, including non-scientific one (as in ancient Egypt, China or Greece [1]), that has tried to establish the relation between facial morphological features and the personality or character of an individual. This possibility was the topic of research in diverse fields such as ophthalmogeometry and physiognomy [2]. Despite the fact that some of these approaches have been dismissed, the recurrent interest in this topic shows that it is still an interesting research question.

Although the accuracy of personality judgments from faces is questionable [3], it is well established that the face plays a central role in the everyday assessments of other people [4]. People agree when they evaluate faces and use these evaluations to infer specific behavioral or interaction intentions. Faces are evaluated rapidly and this process influences social outcomes including but not limited to elections or court room decisions [5]–[7].

In a world characterized by an ever growing amount of interactive artifacts, it is important to develop better human-centric systems that incorporate human communicative behaviors. Natural interaction with machines, one that mimics interactions between humans, is hence an important research goal for computer science that converges with similar interests from other disciplines such as social psychology. The understanding of the social value of objects, including faces, requires the development of engaging interactive systems that act in socially meaningful ways [8]. For this purpose, analysis of facial images has become a major research topic with clear multidisciplinary implications.

For instance in [9], Schlicht et al. studied if rapid evaluation of faces is used in competitive game scenarios to modify decision making. They investigated if people infer their opponent’s style from facial information, and use this knowledge to adjust their betting behavior. The authors used a competitive game scenario (a poker game) to determine if the use of information on judgments of trustworthiness systematically changes wagering decisions, regardless of the feedback on the outcomes. They found that facial information is used to adapt a person's behavior (wagering decisions) in situations where estimation of hidden variables (i.e. playing style) must be done through observable variables. Specifically, they showed that avoidance cues yield bold decisions, whereas approaching cues yield cautious decisions regarding the bet.

Other personality traits seem to have a more permanent effect on the relations and perceptions in social groups. The perception of dominance has been shown to be an important part of social roles at different stages of life, and to play a role in mate selection. Such perceptions positively correlate with dominant behaviors and relational aggression [10]–[13].

If the information on which the evaluation of faces is based could be automatically learned, it could be modeled and used as a tool for designing better interactive systems [14], [15].

The aim of this paper is to study to what extent this information is learnable from the point of view of computer science. Specifically, we formulate the task as a classification problem with the objective of predicting a facial trait judgment. Additionally, a second objective of the study is to find out what information is computationally useful for the prediction task.

To achieve these objectives, we use a machine learning framework and derive a system that captures and interprets facial information in several different ways. Subsequently, via state of the art classification rules, the proposed system learns several trait judgments. Once learned, these models are used to evaluate the system on new, previously unseen examples. That is, the system is able to produce a confidence measure on the most likely trait judgment that could be made by a person, when presented with a new image.

The development of the system consists of two stages: the learning stage, where the models of facial information with respect to the trait judgments are learned from data, and the prediction stage, where trait judgments are produced by the classification rules.

The first stage also attempts to determine which is the best face representation. To this end, we test two approaches: a holistic, appearance-based representation, which encodes all available information about a face, and a structural representation, which encodes exclusively the geometry of the face. The latter approach aims to decrease the amount of information used to describe the face, i.e., the representation is reduced to the relations among a small number of points located either in positions perceived to be perceptually relevant or physically descriptive of the face. In this case, we address the question of the possible relation between components of this structural representation and specific facial trait evaluations. The objective is to establish if there are specific relations and/or points within the face that can be associated with any of the facial trait evaluations.

Regarding the main question of the study, the experiments using a labeled facial data set show that two of the studied traits - dominance and threat can be predicted well beyond chance (over 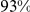 accuracy for this data set) with 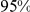 confidence levels. Others can be predicted with accuracy still better than chance (over 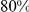 for a 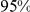 confidence level).

Furthermore, comparison among the techniques used to describe the facial information indicate that, the predictability of facial trait evaluation tends to be more reliable when based on a global representation of the face appearance, than on the information that can be compounded from the structural approach.

With respect to the relations between facial trait evaluation and the facial structure, the experimental results suggest some interesting relations that could serve as starting points for further studies. For instance, there were specific relations between points in the mouth area and perceptions of extroversion.

The paper is structured as follows. In the next section, we review prior findings. The results and the general findings of the experiments are introduced in the subsequent section. Thereafter, the structural and holistic approaches are evaluated and their performance discussed in relation with the proposed objectives. Finally, the Material and Methods section explains in a more detailed manner the data sets, models and experimental framework.

### Related Work

In [16], Oostehof and Todorov realized a series of behavioral studies directed to identifying the basic underlying dimensions of human facial traits evaluation. In their study, they developed a 2D model of face evaluation. The authors gathered unconstrained trait descriptions of an amateur actors face database [12] and clustered them into broad categories. A Principal Component Analysis (PCA) was performed on the linguistic judgments of the traits and two fundamental dimensions were identified. They named these dimensions *Valence* and *Dominance*. They mentioned that the model is applicable to implicit face evaluation where no context is involved. They concluded that valence related cues are related to inferences about harmful/harmless intentions, and dominance related cues are related to perception about the individuals' ability to implement these intentions.

In [17] Brahnam developed a systematic study of what the author called “Physical Personality of the Face”. The author modeled aspects of personal appearance that produce an initial impression of personality in an observer. PCA was used to match human classification of faces along four trait dimensions. In [8], [18], Brahnam & Nanni extended the previous work by including machine learning methods on local face recognition techniques, and expanded the set of traits and the data set of face images. Gabor filters [19] and Local Binary Patterns [20], [21] were used with a pseudo-sliding windows approach as descriptors, and Support Vector Machines [22] and Levenberg-Marquardt Neural Network [23] as decision rules. In both studies, they worked with the program “Faces: The ultimate Composite Picture”, available online, from which they constructed the set of images by either one of two processes: (i) random selection of facial regions (e.g. eyes, noses, lips and jaws) to form a face and subsequently filter those with less real appearance [17]. Or (ii) by carefully generating faces that, according to experts, would exhibit the intended traits [18]. They concluded that machine learning can be used to learn trait predictions, and that it can even outperform individual human annotations.

In [24], Rojas et al. presented a computational system to estimate whether the facial trait evaluation can be automatically learned. They used the information contained in a scarce number of facial points and their geometrical relations as a feature vector and several classification rules. Their findings suggest that facial trait evaluation can be learned by machine learning methods.

In this study, our aim is to find whether appearance or structure information of the face is useful for the prediction of facial trait evaluation. We adopt a classification framework to evaluate visual information cues, using standard machine learning algorithms. In contrast to [8], [18], where the classification method is based on descriptors extracted from sub-images of sliding windows, we tackle this problem from a two different perspectives: a holistic and a structural approach.

Many feature extraction techniques can be applied to the pixel values in order to extract discriminant and invariant descriptors (such as Gabor Jets, PCA or HOG). In that context a holistic approach is the one that takes into account the whole appearance and texture of the face [25]. In this work the holistic approach uses two algorithms that capture facial appearance information in different ways. The first analyzes pixel information via the EigenFaces method [26]; this scheme is based on information theory and intends to find the principal components of the distribution of faces, that is, the algorithm projects the images onto a feature space that spans the most significant variations among the set of images. The second is the robust “Histogram of Oriented Gradients” (HOG) [27], which captures the appearance of the object from the changes in the intensity information of local regions for the entire face; it takes into account the strength and orientation of these changes to generate the global face descriptor.

The structural approach uses only the locations of specific fiducial facial points, which are considered to be salient from a perceptual point of view. These landmarks are combined in different ways to form a geometric descriptor of the face.

Finally, both approximations are validated through a bank of state of the art machine learning classifiers to assess their general performance and the validity of the results.

## Results

The problem is tackled from the perspective of a classification task. We use machine learning techniques to evaluate the proposed two hypothese: first, whether the automatic prediction of facial trait judgments can be performed using a structural or holistic approach, and second, verify whether there are points in the structure or relations in the geometric descriptor that can be related to any of the analyzed trait judgments (for details on the traits analyzed see the *Materials and Methods* *– Data*).

The results presented in this section were computed as follows. First, we obtained a descriptor for each facial image, using the proposed feature extraction techniques (see *Materials and Methods* *- appearance/geometric descriptor*). Then, a subset of the samples (training set) has been selected and used to train the models for each trait on each one of the descriptors. Each model consists of a properly trained classifier from the proposed bank of machine learning techniques. The resulting accuracies depicted in tables 1, 2 and 3 of this section are the output of applying the classification models to the remaining samples (test set). In the experiments, 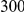 images, from the synthetic database mentioned in [16], were used to train the models. Details on the ground truth data generation, the statistical validation protocol, and the estimation of the classifiers parameters are presented in the Materials and Methods Section.

**Table 1:** Mean accuracy and (confidence interval) for the Structural Approach.

Trait	Attractive	Competent	Trustworthy	Dominant	Mean	Frightening	Extroverted	Threatening	Likable
**GB**	82.52 (6.5)	68.81 (8.7)	75.59 (8.0)	87.52 (7.1)	76.15 (6.0)	76.98 (7.6)	83.11 (6.4)	90.86 (4.4)	72.55 (8.9)
**SVM**	75.45 (5.5)	72.27 (9.0)	77.95 (8.9)	87.09 (6.1)	82.25 (5.0)	80.74 (7.6)	91.42 (5.7)	87.52 (5.2)	70.45 (8.7)
**BTree**	63.51 (7.0)	65.77 (8.1)	75.05 (8.7)	74.48 (6.9)	71.85 (7.1)	71.85 (8.9)	64.93 (9.8)	76.98 (4.3)	52.27 (9.8)
**5nn**	66.58 (5.8)	63.81 (7.6)	70.47 (7.5)	79.46 (6.3)	71.15 (7.0)	67.84 (5.4)	75.18 (9.6)	81.69 (6.2)	62.57 (8.8)
**Parzen+RS**	75.59 (9.0)	62.70 (12.0)	67.14 (10.2)	77.79 (8.7)	67.68 (8.4)	64.91 (5.8)	70.47 (9.2)	71.42 (6.2)	75.61 (7.7)

**Table 2:** Mean accuracy and (confidence interval) for the EigenFaces method.

Trait	Attractive	Competent	Trustworthy	Dominant	Mean	Frightening	Extroverted	Threatening	Likable
**GB**	46.87 (8.1)	60.23 (10.3)	57.97 (9.0)	84.89 (6.7)	65.16 (7.9)	75.99 (8.1)	57.30 (9.4)	73.24 (8.4)	50.47 (10.1)
**SVM**	63.54 (9.3)	69.91 (8.7)	59.50 (8.0)	93.22 (5.6)	74.89 (6.0)	80.72 (8.0)	62.57 (10.8)	82.55 (5.5)	59.64 (8.3)
**BTree**	57.00 (8.0)	60.63 (9.3)	52.84 (6.8)	89.89 (6.0)	67.82 (7.7)	54.21 (9.2)	50.07 (8.2)	77.41 (6.4)	54.77 (10.3)
**5nn**	62.14 (8.2)	61.17 (8.2)	56.04 (9.5)	89.05 (6.0)	73.24 (8.0)	66.01 (7.7)	52.84 (9.0)	77.27 (5.0)	47.27 (8.5)
**Parzen+RS**	67.41 (6.9)	63.67 (11.2)	66.44 (8.2)	77.79 (7.3)	66.28 (8.8)	64.08 (6.4)	70.05 (9.9)	83.36 (6.3)	76.85 (8.6)

**Table 3:** Mean accuracy and (confidence interval) for the HOG method.

Trait	Attractive	Competent	Trustworthy	Dominant	Mean	Frightening	Extroverted	Threatening	Likable
**GB**	75.02 (6.0)	69.05 (8.6)	79.46 (6.3)	96.67 (3.0)	84.89 (5.8)	75.05 (8.0)	90.02 (5.0)	94.46 (3.4)	70.32 (10.8)
**SVM**	81.13 (6.0)	81.55 (6.7)	91.13 (4.4)	96.67 (3.0)	88.09 (5.9)	87.25 (6.3)	85.59 (6.4)	97.79 (2.4)	83.49 (8.6)
**BTree**	66.85 (7.9)	55.47 (6.3)	73.92 (8.7)	84.73 (6.1)	77.68 (8.6)	72.27 (8.7)	72.95 (6.8)	84.21 (7.3)	74.21 (7.7)
**5nn**	73.81 (5.7)	68.54 (6.1)	78.24 (5.9)	93.06 (4.3)	81.28 (6.7)	78.38 (7.5)	77.55 (8.3)	91.82 (5.5)	76.71 (7.6)
**Parzen+RS**	75.07 (7.8)	66.35 (11.4)	70.13 (9.0)	81.68 (7.8)	70.33 (8.4)	67.72 (5.9)	73.77 (9.4)	81.26 (6.1)	80.04 (8.0)

The two variables involved –appearance and structure– were analyzed separately. For the holistic approach the images of the faces were projected on a reference image shape to normalize the structure, thus measuring only appearance (see *Materials and Methods* *, appearance descriptor* for further information). In the case of the structural approach, only the spatial coordinates of the fiducial facial points are considered, discarding any appearance information.

The mean accuracy results shown are computed using a 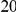-fold Cross Validation framework, and are complemented with the corresponding figure for the confidence interval, for a 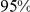 confidence level “ shown in brackets (see *Materials and Methods* *– Data*).

### Structural Approach

Table 1 shows the performance of the geometric descriptor for all the classification rules with respect to each trait. “Dominant” and “Threatening” score well above chance (over 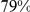) for at least 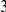 of the classification rules; “Trustworthy”, “Extroverted”, “Frightening”, and “Mean” also perform better than chance (over 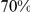) for at least 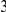 classification rules.

### Holistic Approach

Table 2 shows the performance of the classic EigenFaces method (see section *Materials and Methods* *- appearance descriptor* for details) for all the classification rules with respect to each trait. In this case, “Dominant” and “Threatening” score well beyond chance for all of the classification rules, and “Mean” and “Frightening” have a good accuracy for at least 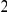 rules. All the other traits show a near chance prediction scores, suggesting that this method is not well suited to render an appropriate descriptor for the classification task, when presented with appearance only data as the one used in this case. In the light of these results, we extended the holistic descriptors with the use of the current state of the art HOG feature extraction algorithm.

**Figure 1:**
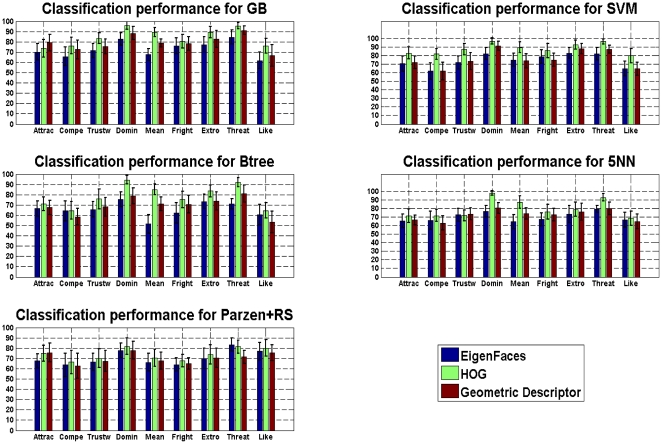
Mean performance as a function of traits. Comparison between the implemented classification rules (vertical lines represent the confidence intervals).

Table 3 shows the performance of the HOG method (see section *Materials and Methods* *appearance descriptor* for implementation details). In this case, all traits except “Competent” exhibit high accuracy for all the classifiers. “Dominant”, “Threatening”, and “Mean” exhibit the highest and most consistent scores for all 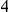 classification rules with accuracy higher than 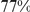 for all the classifiers. Figure 1 summarizes the performance for the three methods implemented per classifier for all the traits. It can be seen that the HOG method performs slightly better than the other two, for at least 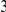 of the classification rules. This suggests that the holistic approach is better suited to handle the prediction task. With respect to the performance per trait, it can be seen that “dominant”, “threatening”, and “mean” are learnable regardless of the descriptor method employed.

**Figure 2:**
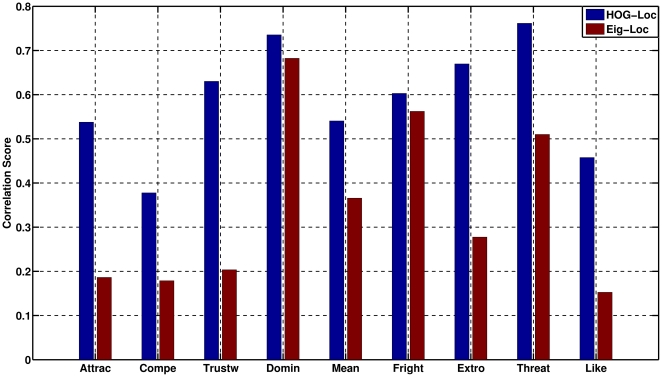
Correlation between Holistic and structural methods. The correlation was done over the predicted classes per trait for the SVM classifier.

In light of these results, further analysis was done to find out whether the information conveyed by a holistic representation is complementary to the one conveyed by a structural one. In this analysis, we took the labels predicted by the appearance and geometric descriptors and correlated them to test if the same images were labeled in the same way by the classifiers. Figure 2 shows the correlation scores for the pairs HOG-Geometric and EigenFaces-Geometric descriptors, for all the traits.

It can be seen that the correlation is high for dominance, suggesting that regardless of the method used, this trait judgment can be accurately predicted.

On the other hand, the low correlation of the EigenFaces-Geometric descriptor pair suggests that there is little relation in the way the information is described by the two methods. The values in figure 2 resemble those in table 2 for the Eigenface method, where in contrast to the HOG method, the prediction capabilities for traits such as trustworthy are weak.

In the case of the HOG-Geometric descriptor pair, the correlation scores are close to 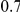 in the “trustworthy”, “dominant”, “extroverted”, and “threatening” judgments. In contrast, judgments of “competence” and “likeability” have a low correlation coefficient, which is consistent with the prediction capability of the geometric descriptor on these traits as shown in table 1. This suggests that the trait judgment information is encoded differently for each trait, and that both methods may capture that information in a different way, which may make them more suitable for specific traits. Nonetheless, according to these same results, the characterization done by the HOG method seems to be general enough to predict, with a good level of confidence, the trait judgments.

### Descriptors and Traits

This section presents the experiment that aims to establish if there are specific regions within the face that can be associated with any of the facial trait evaluations. The experiment was performed using the geometric descriptor (see *Materials and Methods* *- Geometrical Descriptor*) and the ground truth labels for each trait. We computed the normalized correlation between each feature in the geometric descriptor and the ground truth labels, trying to identify the most significant regions for facial trait judgments evaluation, by counting the amount of times a given point is used to compute the feature in the geometric descriptor.

Results reveal that there is correlation between the geometry of several points and the perception of attractiveness and extroversion. For the first, the area around the eyes shows a clear correlation with the trait judgment; the alignment, size, and distance between the points extracted from the region of the eyes are correlated with that trait judgment. Furthermore, there are relations between the eyes and the lips, and between the eyes and the nose that show correlation to that trait judgment as well.

In the second case, the perception of extroversion is correlated with the mouth area, specifically with the size of the lips. There is also a relation between the mouth and the chin, in terms of spatial distribution, and the judgment of extroversion. These relations are in concordance with the results presented in [28], where cues in these areas are related to both the personality measures of extroversio and facial trait judgments.

**Figure 3:**
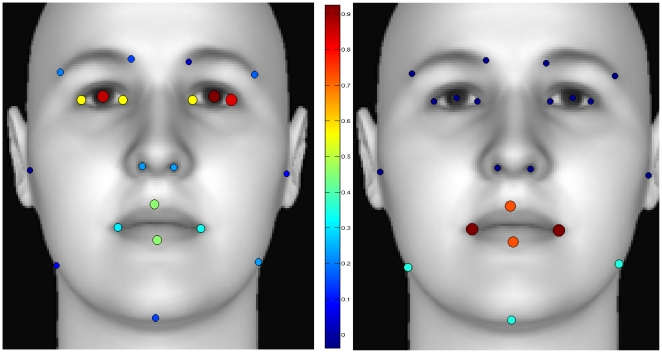
Correlation of facial points and facial trait evaluations. Left: Attractive, Right: Extroverted. The size and color of the circles is proportional to the number of times a given point is used in a specific feature of the geometric descriptor. Small dark blue circles represent low correlation.

Figure 3 shows the locations of the points that correlate with the mentioned traits. Color and size coded circles are used to denote the correlation between a given point location and a certain facial trait prediction. The number of times a point is used to compute a feature in the geometric descriptor is normalized and used as a measure of the radius, and as reference of the color (using a Jet-Colormap, where low values are coded in dark blue, and high values are coded in dark red) of the circle.

No other clear relations between the geometric descriptor and other trait judgments were found.

This analysis was then applied to the possible correlations between the geometric descriptor and the labels projected on the first two principal components, Valence and Dominance, of a PCA of all trait judgments. This is based on the results of Oosterhof and Todorov in [16], where they found that broad categories of traits could be approximated by judgments on these two dimensions.

In this analysis, relations between the upper half of the face, specifically the eyes and eyebrows areas, and the first principal component were found. Weaker relation between the first principal component and the nose and chick bones was also found.

In general, angles were more correlated with the trait judgments than distances (almost 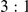 proportion) with the trait judgments. These relative positions of the facial elements can be understood as a measure of symmetry or facial harmony.

## Discussion

We studied the problem of determining the prediction capabilities of an automatic system with respect to the task of facial trait judgments. We tackled the question from two perspectives, a holistic and a structural approach, and used machine learning techniques to answer the question on the automatic predictability.

We implemented two different methods for the holistic approach, namely EigenFaces and HOG methods, and one method for the structural approach. The former describes the images in terms of the appearance information, and the latter uses the relations among a few salient points in the image of the face to describe it.

The classification was done using state of the art classifiers. Five algorithms were employed: GentleBoost as an example of additive method, Support Vector Machine with a Radial Basis function kernel as an example of the non-linear classifiers, K-Nearest Neighbor as an example of a non-parametric classifier, Parzen Windows with RandomSubspace, and Binary Decision Trees. The evaluation of the system was performed by using a 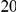-fold cross-validation strategy, and the results were supported by the confidence intervals computed for a 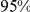 confidence level.

The results of the experiment confirm that facial trait evaluation from neutral faces can be computationally learned. More specifically, three traits “Dominant”, “Threatening”, and “Mean” can be learned by an automatic system well beyond chance. Furthermore, it was observed that both facial representations are complementary to one another, and that each trait was encoded differently suggesting that there are representations better suited for specific traits.

Regarding the comparison between the holistic and the structural approaches, the results show that a more consistent and reliable prediction can be obtained when considering the appearance of the face. Nevertheless, this does not necessarily marginalizes the prediction capability of the structural approach. As can be seen from figure 1, its performance is quite close to that of the HOG method, although the structural method uses a simpler representation.

In summary, we experimentally validated the computational prediction capabilities of facial trait judgments. We have shown that all the analyzed trait judgments can be predicted. Furthermore, at least three judgments exhibit prediction accuracy beyond 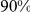. This prediction capability was found to be more strongly related to the holistic facial representation than to the structural relations employed.

## Materials and Methods

### Data

In this study, we used the behavioral data obtained by Oostehof and Todorov in [16]. In this section, we briefly review the procedure to determine which traits could be evaluated and how these traits lead to the generation of a two dimensional model of the facial trait evaluation.

In a first step, the facial trait dimensions were identified in an experiment involving 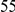 undergraduate students from Princeton University. Each student wrote an unconstrained description from a set of 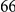 standardized faces from the Karolinska [29] amateur actors face database. 1134 descriptions were collected, and two researchers independently classified the attributes from the descriptions into broad categories (discrepancies were solved by a third party). The researchers classification of the unconstrained descriptions resulted in 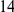 selected categories.

In a second step, the 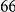 faces were rated on a continuous scale by a separate group of 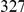 participants based on their first impression; faces were presented three times in separate blocks. The question How [trait] is that person? was presented altogether with the centered face, and a response (in the range 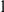 to 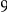) was to be given.

A data-driven model for the evaluation of facial trait inferences was built. A Principal Component Analysis resulted in two prevalent orthogonal dimensions accounting for over 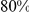 of the data variance (according to [16], the third PC accounted for less than 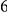
% of the data variance and had no clear interpretation); these dimensions were denominated *valence* and *dominance*, respectively.

In a third step, a synthetic face database was generated using the FaceGen software [30]. The software used a statistical model based on a large set of 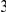-D lasers scans of real faces, where the shape of each face is represented as a mesh of 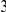-D vertices. A Principal Component Analysis was performed on these coordinates preserving the 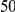 components that account for most of the data variance. Faces were randomly generated using this model, where small changes on each PC coefficient produce holistic changes on the vertex coordinates of the face image. 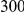 Images were randomly generated bounding the software to generate Caucasian faces with neutral expression.

Subsequently a new set of dimensions (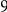) was used to rate the faces and this is the set used in the current paper. This new set was used because a larger number of faces was rated and the results obtained for these faces and dimensions were similar to those of the original set of faces and traits [16].

Using the synthetic images data set (available under request at http://webscript.princeton.edu/~tlab/databases/database-1-randomly-generated-faces/) and the trait labels provided with it, we evaluated the following traits: Attractive, Competent, Trustworthy, Dominant, Mean, Frightening, Extroverted, Threatening, and Likable – these traits presented a high reliability (between 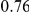 and 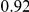 Cronbach's alpha) of interrater agreement. For further details see table S1 or see [31]. In our study, we used this synthetic images data set and the trait labels provided with it. We experimented on the complete set of trait dimensions and not only on the two prevalent orthogonal dimensions found in [16] given that the projections lose information that is important for specific judgments (e.g. in the case of the synthetic data set, the first PC accounts for 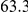
% of the variance only, and the second accounts for 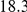
% only leaving almost 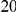
% of unaccounted information – for further details on the quality of the projections see figure S1).

**Figure 4:**
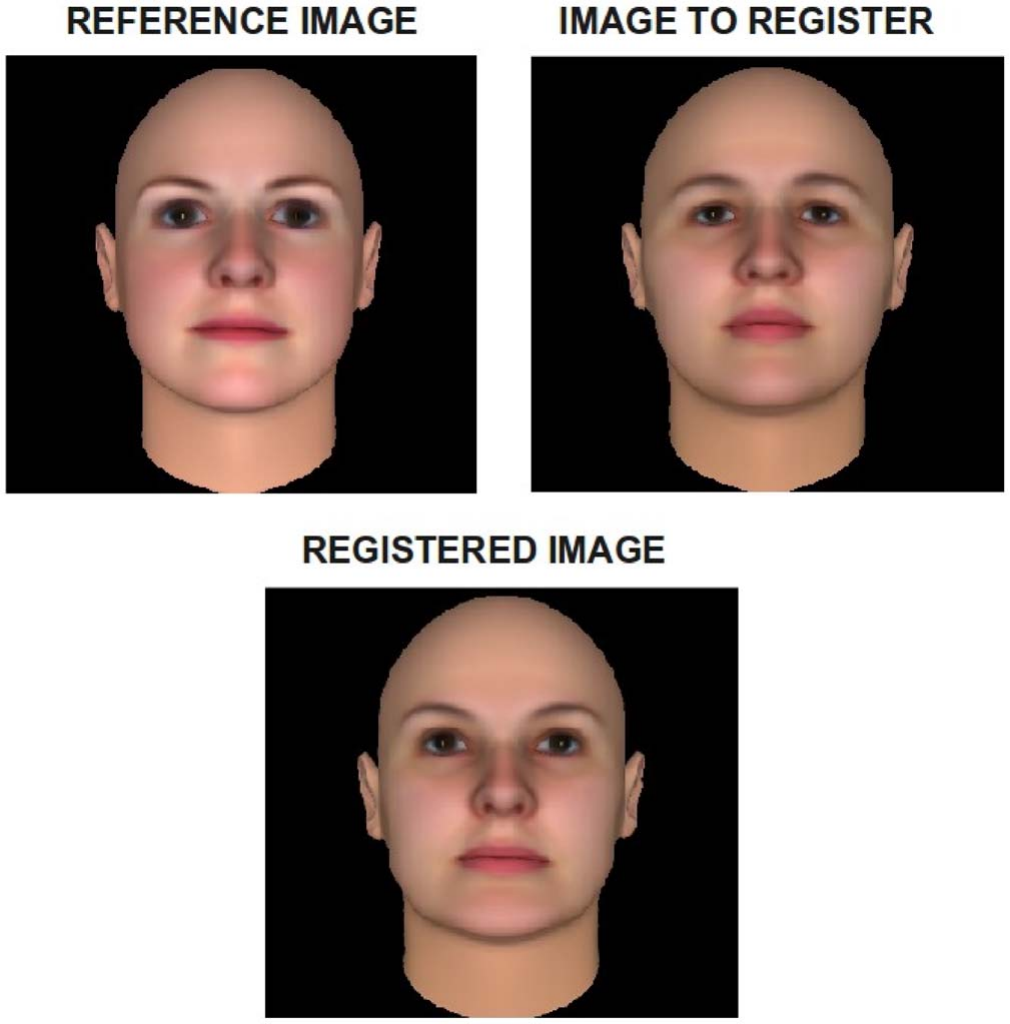
Controling the variables examined in the experiments. The appearance variable is isolated controling the structure part of the face by projecting the images of each face to a reference face image.

Given that the holistic approach was used to describe appearance, variations on structural information needed to be standardized. To do this, all the faces of the data set were projected onto a reference image shape. This image was chosen to be the closest to the mean face to balance the amount of deformation the faces would suffer. The projection process was done by means of an affine based registration and data fitting of the 2D intensity data, using a b-spline grid to control the process. We used the implementation developed by Dr. Dirk-Jan Kroon of University of Twente, available on the Mathworks file exchange web site. Figure 4 shows the reference image, the image to project and the resulting image projected onto the reference shape.

On account of the ranking of each trait (in a range 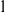 to 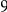), the problem had to be adapted to a binary one. Thus, it was necessary to sort each trait according to its rank, and generate the *class* (high score for the trait) and *no class* (low score for the trait) subsets from the highest (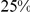) and lowest (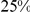) ranking elements respectively. This separation of the data through the binarization of the scores intends to reduce the noise product of mislabeled samples or outliers.

Because of the small sample size resulting from the previous procedure, for each classifier of the bank, the error rate was estimated with a N-fold cross-validation scheme. This is a way of splitting a data set where *(N-1)/Nth* of the data are used for training and the remaining *1/Nth* used for testing, with *N-1* subsequent non-overlapping iterations. As mentioned in the *Results* section, for our experiments with the synthetic data set, 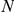 was set to 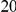.

The results shown for the performance are given with a confidence interval (shown in brackets in tables 1, 2 and 3) for a 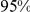 confidence level, computed as:
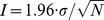
(1)with 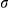 being the standard deviation of the results, and *N* the number of folds performed in the Cross Validation framework used.

### Geometrical Descriptor

We have used the 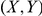 coordinates of a set of predefined facial salient points, and generated the geometric descriptor derived from three types of relations.

**Figure 5:**
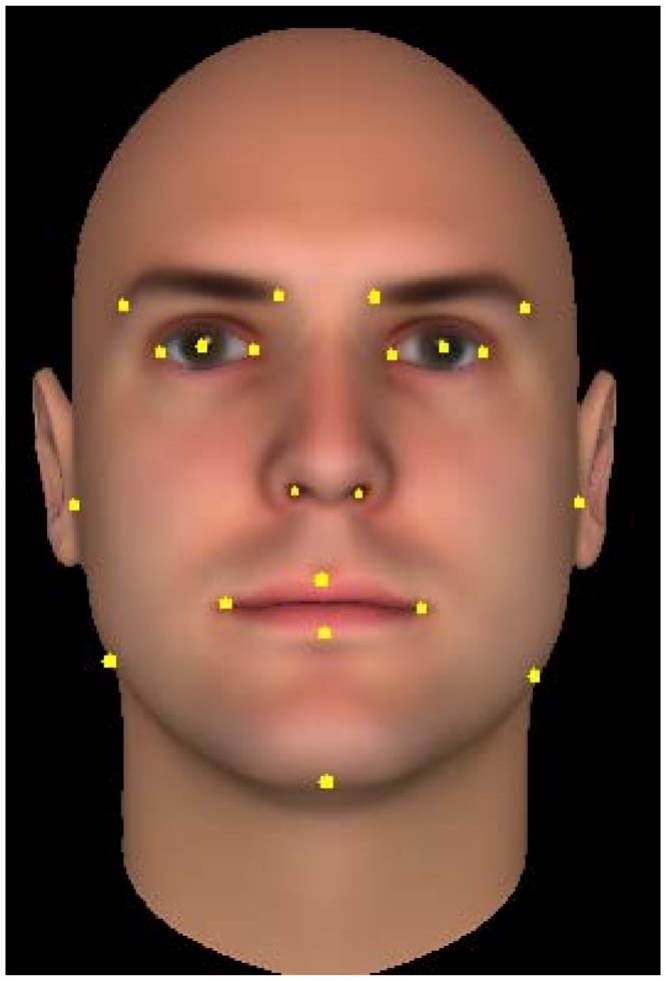
Points used to describe the facial structure.

Twenty one predefined point locations 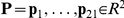 from each face are manually marked and the mean coordinate values 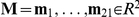 of the database are computed (figure 5). The selection of these points was partly based on the fact that they represent the most commonly used in applications of facial and gesture analysis [32], [33]. Using this information a 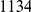-dimensional structural feature vector of the face is computed as follows:

1. The first 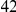 values of the descriptor consist of the difference of each point 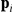 to its corresponding mean 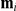 (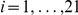). In order to extract more information on the difference the computation is done in polar coordinates, and the values for angle and radius are stored.
2. The second set encodes the spatial relations between each salient point 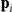 of the face and all the points of the mean face image 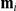 (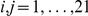) in terms of radius and angle, hence generating a 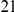
×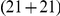, 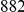 dimensional sub vector.
3. The third set encodes the intra face structural relationships, and consists of 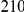 values with the euclidean distances of each point 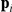 to all the other points in the same image 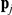 (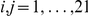).

### Appearance Descriptors

#### EigenFaces

The EigenFaces method [26] has been successful in different face classification tasks. Essentially, the method is based on applying the PCA technique to the normalized high dimensional facial samples.

**Figure 6:**
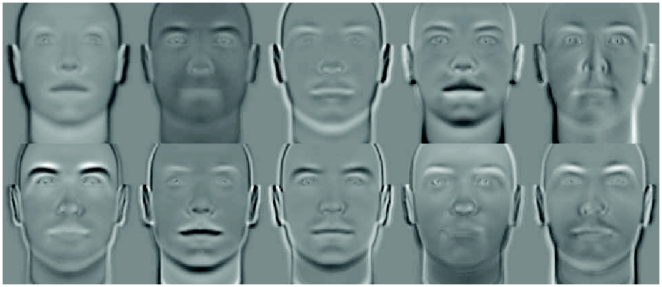
EigenFaces Method. Ten first Principal Components of the Dataset.

For the experiment, we cropped the images to a size of 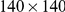 pixels. The PCA was applied over the vectorized images, preserving 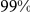 of the information. Figure 6 depicts the first 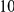 principal components for the synthetic database.

In order to verify the separability of the dataset with respect to the appearance, we have projected the two traits that showed the higher prediction capabilities in our experiments. We used the PCA technique to reduce the pixel data information to only two dimensions. Using this approach and for visualization purposes, each facial picture was projected to a 2D feature space using the first two EigenFaces as bases.

**Figure 7:**
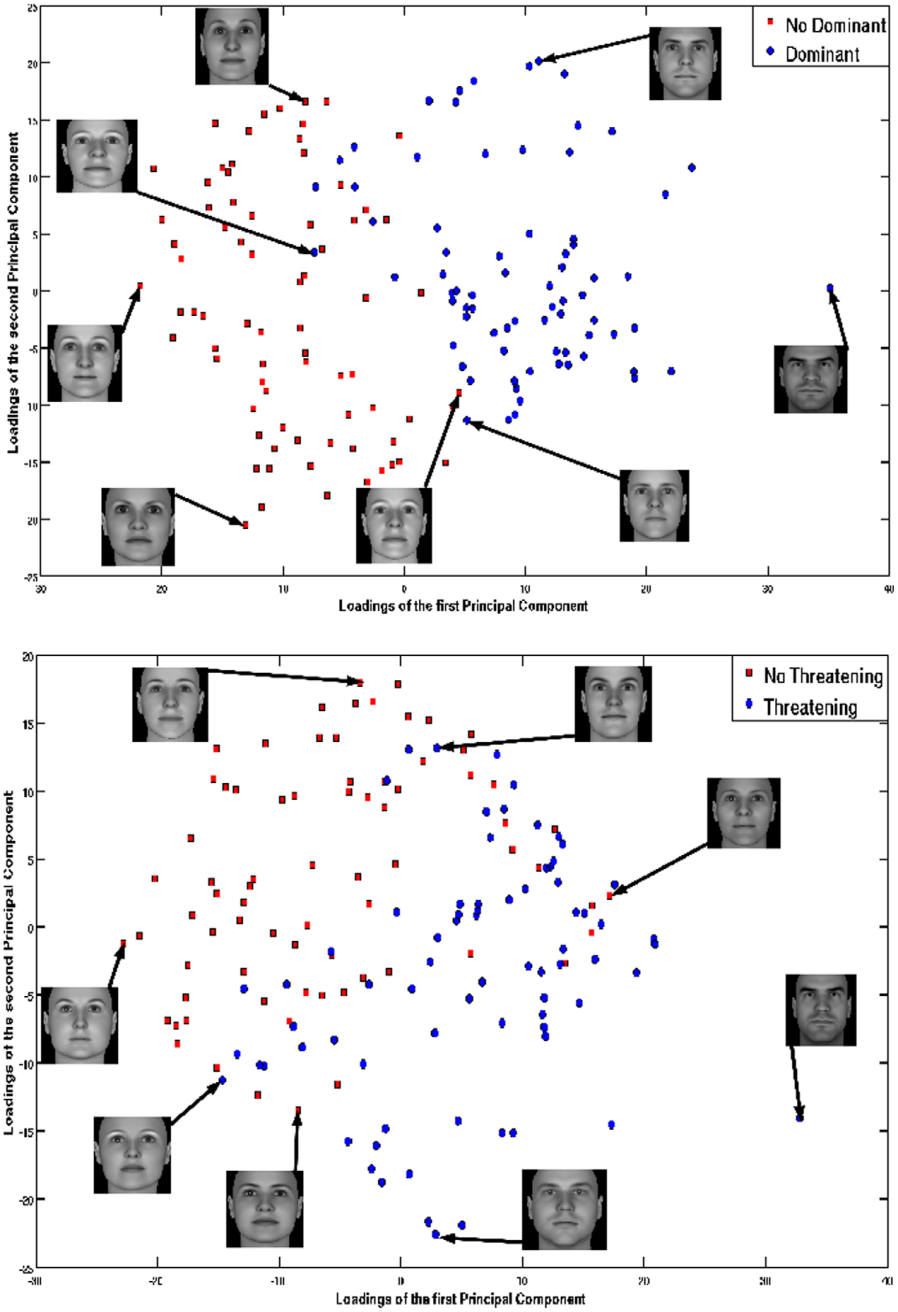
Scatter Plot of the projection of the training set on the first two Principal Components. The traits with highest prediction scores in the experiments - (Top) Dominance and (Bottom) Threatening, are shown.

Figure 7 depicts the training set projected on the first two Principal Components for the judgments of Dominance and Threat respectively. We plot the prevalent orthogonal dimensions proposed in [16] using blue for the dominant/threatening and red for the non dominant/non threatening samples. Thumbnails of the samples in the boundaries of each subset (class/no class), are shown for both Principal Components. The spatial distribution of the samples using the EigenFaces approach highly correlates with our intuitive idea of dominant/threatening.

#### Histogram of Oriented Gradients - HOG

This method was developed with the purpose of general object recognition, where the appearance and shape are the targets of the characterization, as mentioned in [34]. The basis of the algorithm are the edge orientation histograms; the strength of the technique lays in the division of the image in groups of pixels called cells over which the histograms are computed, and on the overlapping normalization of the blocks (groups of cells).

Our implementation of the HOG method is applied to the entire object, hence obtaining a unique descriptor, that is, the image containing the object is divided into a uniform grid of cells and a histogram is computed for each cell. The illumination normalization is performed by grouping cells in blocks to avoid local changes in illumination. These blocks take overlapping cells according to a user defined parameter, thus replicating the presence of a cell-histogram in the final descriptor, but normalized to a different block. Thus, we define the HOG approach as holistic due to the way the descriptor is built using overlapping normalizing blocks all across the object. Notice that neither separate HOG descriptors for separate regions are computed, nor any geometric relationship among cells or blocks to build this descriptor is used (which could be considered as a local approach as in [35], where objects are characterized by its parts and their location in the object).

**Figure 8:**
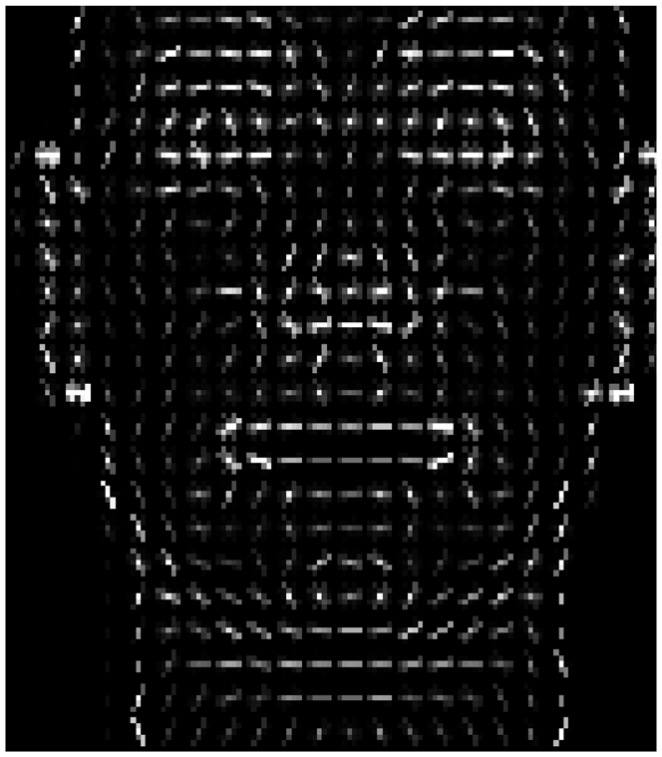
Histogram of Oriented Gradients of the face.

Finally, we extract a concatenation of the histograms computed at the different cells. The current implementation uses an unsigned gradient, that is, the orientation bins are evenly spaced over 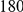 degrees. 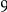 bins quantize the orientation histograms in ranges of 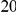 degrees per bin. The final descriptor is built in a region of interest of 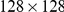 pixels, by concatenating the block normalized cell histograms. Each cell is 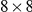 pixels and each block is 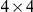 cells, with an overlapping factor of 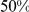. Figure 8 illustrates the descriptor product of applying the HOG method to a face.

The algorithms for the generation of the HOG descriptor and the Geometric descriptor, as well an implementation for the PCA method can be downloaded from http://www.cvc.uab.es/~davidm/code/Descriptors.zip.

### Machine Learning Methods

The evaluation of the study was done by using a bank of classifiers composed of state of the art methods. Four were selected as examples of the different types of approaches:

- GentleBoost [36] is a variation of the original boosting method. It modifies the update of the strong classifier changing the way it uses the estimates of the weighted class probabilities. The current implementation is trained over 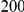 iterations using *stumps* as weak classifiers.
- Support Vector Machine [22] is an example of non-linear classification rule. In our experiments the SVM used as kernel the Radial Basis Function [37], and the parameters are computed via an iterative optimization subroutine from the toolbox, using a subset of the training data, which is extracted by the subroutine itself.
- Binary Decision Trees [38] is an acyclic graph used to represent a Boolean function. It is a data structure that consist of a decision node that labels the Boolean variable and possesses two child nodes that correspond to each variable state. In the current implementation, the parameters for pruning and splitting criterion are optimized by the library routine itself.
- K-Nearest Neighbor [22] is an instance based classification algorithm, where the results of new instances are labeled based on the majority of the k most proximal training samples. Prior analysis suggested that the appropriate value for 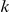 was 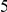 for these studies.
- Parzen Window
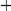
Random Subspace; Parzen window is an instance based density estimation where kernel functions (windows) determine the contribution of the observations falling inside the window; Random Subspace is a method where several learning machines are trained on subsets of the feature space which are randomly chosen. The final model output can be a combination of the outputs of the trained classifiers, in our case, is a simple majority vote.

The implementation of the GentleBoost classifier used is publicly available at Antonio Torralba's web site [39]. The implementations used for the SVM, the binary decision tree, the kNN, and the Parzen-Window classifiers are “off-the-shelf” routines from the PRTools [40] and the PRSD Toolbox [41]. These routines contain the appropriate parameter optimization subroutines and evaluation functions, and allow for a plug-and-play use of the methods. The use of standard classifiers allows us to apply the prediction capabilities of our approach to new unseen samples.

**Figure 9:**
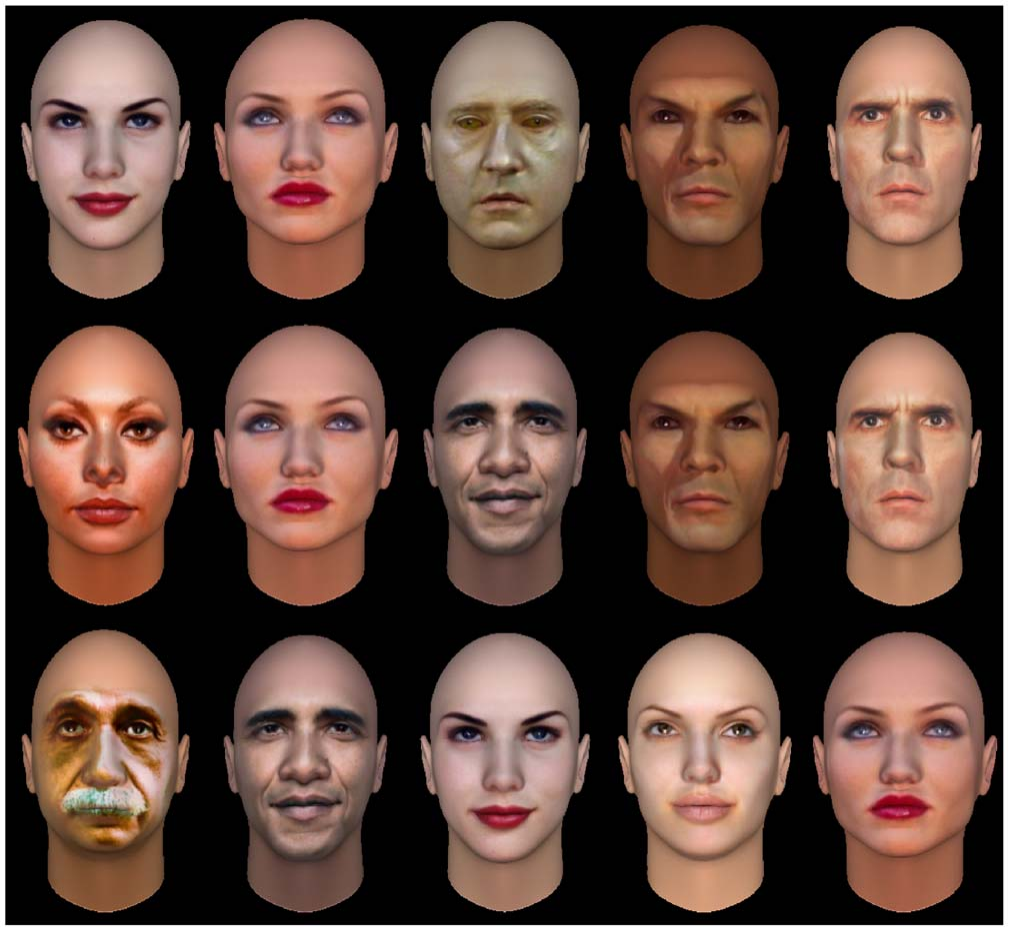
Images of public figures tested by the prediction system. The faces were projected on the same synthetic portraying system used in the study. Images are sorted in increasing rank order from left to right, by Dominance (top row), Threatening (middle row) and Attractiveness (bottom row).

Although this is not the main goal of this evaluation paper, we performed a proof of concept experiment using a gallery of celebrity images and the FaceGen Software. Images of famous public characters (projected on the same synthetic system used in the study) are shown as illustrative examples of the prediction capabilities of the system. The results of the classifiers can be usually interpreted as a continuos confidence value, or degree of support for the classification task, rather than a simple binary label. We use this support to rank the image gallery. Figure 9 depicts the results of the prediction for the traits that have the highest scores in the classification task (Dominant, Threatning and Attractive), which for an unseen set of images produces a prediction that we think of as highly consistent with the idea of attractiveness.

## Supporting Information

Figure S1 Scatter plots of the projections of judgments of the nine traits on the first two principal components derived from a PCA of the traits. It can be seen that each trait projects differently, in the case of dominance projects well to the second PC, where mean and threatening do not project that well hence using the information of each trait allows learning the specific features that make each trait unique.
(PDF)

Table S1Inter-rater agreement and reliability of nine social judgments of emotionally neutral faces for the 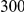 synthetic faces images. Raters (n) were asked to make judgments of 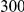 randomly generated faces on a scale from 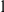 (not at all [trait term]) to 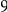 (extremely [trait term]).(PDF)
