# Supplementary figures and images for: Automatic Prediction of Facial Trait Judgments: Appearance vs. Structural Models
Source: PLoS One. 2011 Aug 17;6(8):e23323. doi: 10.1371/journal.pone.0023323 (PMC3157350; doi:10.1371/journal.pone.0023323)

Figure §1.

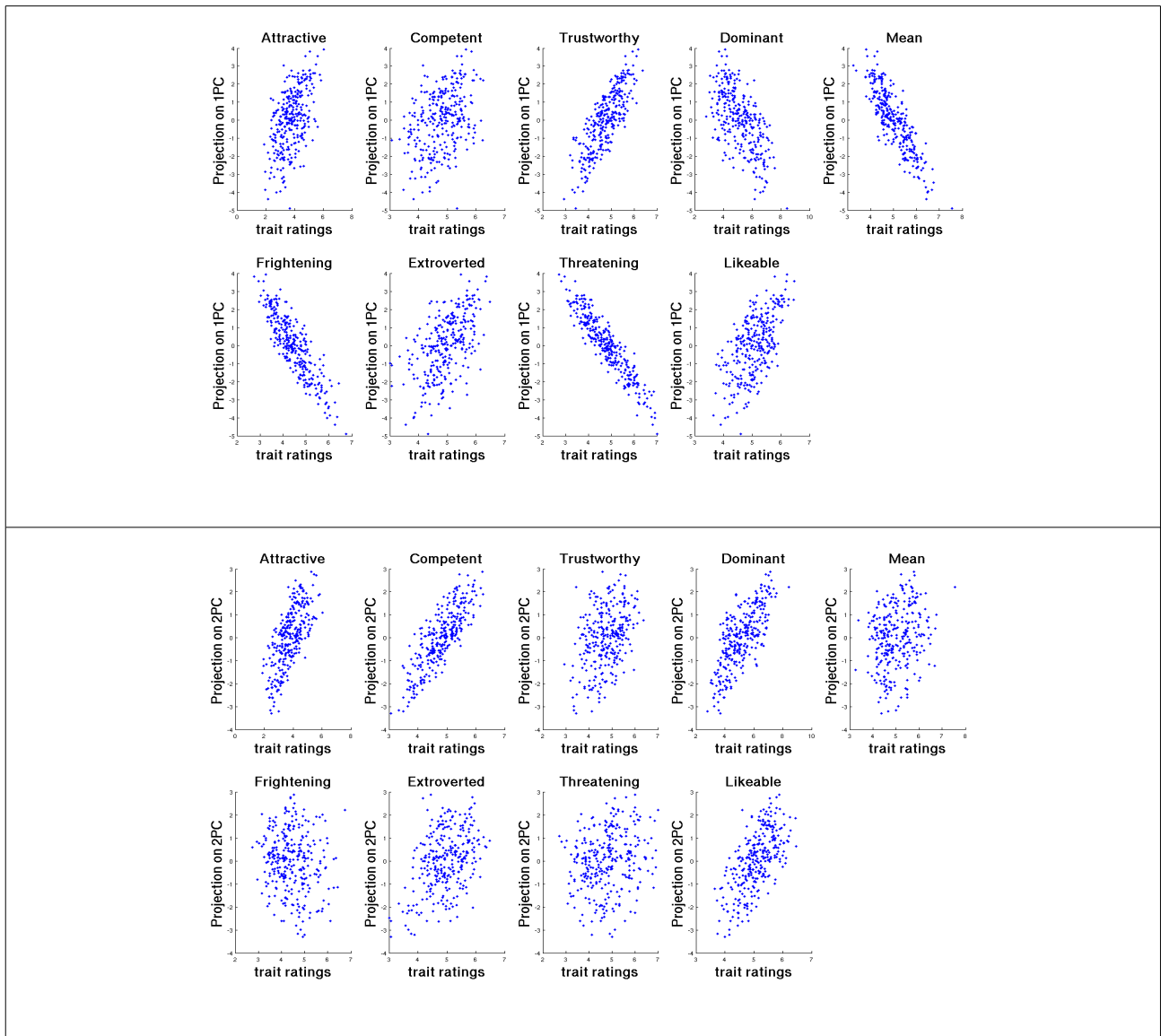

Supplement: Figure S1 — Scatter plots of the projections of judgments of the nine traits on the first two principal components derived from a PCA of the traits. It can be seen that each trait projects differently, in the case of dominance projects well to the second PC, where mean and threatening do not project that well hence using the information of each trait allows learning the specific features that make each trait unique. (PDF) [file pone.0023323.s001.pdf]
