# Supplementary material for: Automatic Prediction of Facial Trait Judgments: Appearance vs. Structural Models
Source: PLoS One. 2011 Aug 17;6(8):e23323. doi: 10.1371/journal.pone.0023323 (PMC3157350; doi:10.1371/journal.pone.0023323)
Supplement: Table S1 — Inter-rater agreement and reliability of nine social judgments of emotionally neutral faces for the synthetic faces images. Raters (n) were asked to make judgments of randomly generated faces on a scale from (not at all [trait term]) to (extremely [trait term]). (PDF) [file pone.0023323.s002.pdf]

Table §1.

Judgment	Number of raters (n)	Inter-rater agreement (r)	Reliability (α)
Dominant	23	.36	.92
Threatening	21	.26	.87
Attractive	35	.23	.91
Frightening	28	.17	.84
Mean	27	.17	.83
Trustworthy	29	.15	.81
Extroverted	33	.14	.84
Competent	44	.11	.84
Likeable	31	.10	.76

Inter-rater agreement and reliability of nine social judgments of emotionally neutral faces for the 300 synthetic faces images. Raters (n) were asked to make judgments of 300 randomly generated faces on a scale from 1 (not at all [trait term]) to 9 (extremely [trait term]).
